# Globus Pallidus Internus Deep Brain Stimulation Using Frame-Based *vs*. Frameless Stereotaxy in Dystonia: A Single-Center Experience

**DOI:** 10.3389/fneur.2021.643757

**Published:** 2021-06-29

**Authors:** Roberto Eleopra, Sara Rinaldo, Grazia Devigili, Massimo Mondani, Stanislao D'Auria, Nico Golfrè Andreasi, Miran Skrap, Christian Lettieri

**Affiliations:** ^1^Parkinson's Disease and Movement Disorders Unit, Fondazione Istituto di Ricovero e Cura a Carattere Scientifico (IRCCS) Istituto Neurologico “Carlo Besta”, Milan, Italy; ^2^Neurosurgery Unit, “S. Maria della Misericordia” University Hospital, Udine, Italy; ^3^Neurology Unit, “S. Maria della Misericordia” University Hospital, Udine, Italy

**Keywords:** deep brain stimulation, dystonia, frame-based stereotaxy, frameless stereotaxy, functional neurosurgery

## Abstract

**Objective:** Bilateral globus pallidus internus deep brain stimulation (GPi-DBS) is an established and effective therapy for primary refractory dystonia. However, the comparison of frameless *vs*. frame-based DBS surgery technique is still controversial. This retrospective study aims to compare the clinical outcome of two GPi-DBS surgical techniques for patients affected by primary generalized or multi-segmental dystonia.

**Methods:** For lead's stereotaxic placement, 10 patients underwent frame-based surgery and the other 10 subjects DBS surgery with a frameless technique. Clinical features were evaluated at baseline and 6 and 12 months after surgery by means of the Burke–Fahn–Marsden Dystonia Rating Scale.

**Results:** Frame-based GPi-DBS and frameless stereotaxic group revealed a comparable clinical outcome with no surgical complications.

**Conclusions:** Frameless technique is safe and well-tolerated by patients and showed similar effectiveness of the frame-based stereotaxic surgery during GPi-DBS for primary dystonia. Notably, it could be a valid alternative solution because of the great advantage in improving the patient's discomfort during awake surgery.

## Introduction

Deep brain stimulation (DBS) is a well-established therapy for several movement disorders, and bilateral globus pallidus internus (GPi) stimulation is used as an effective and relatively safe treatment for different forms of medically refractory dystonia.

Primary generalized, segmental, or multi-segmental dystonias are reported to have the best postoperative outcomes, although the benefits for secondary dystonia are still a matter of debate (1–12).

One of the most important key issues for successful DBS surgery is the lead's placement into the target nucleus: an incorrect positioning may result in ineffective symptom control and adverse effects related to electrical stimulation. Lead' misplacement has been considered as one of the major reasons for patient's discomfort or even DBS failure (13–15). To improve the lead's placement, detailed imaging techniques and intraoperative microelectrode recording (MER) are performed to identify the right target localization during surgery (16).

Traditionally, DBS surgery is performed using frame-based stereotaxy, but, in recent years, frameless techniques are also available.

Frameless stereotaxy combines modern computer image-guidance technology and an advanced navigation system. The skull-fixed frameless system provides a highly stable platform during several hours of surgery without a rigid frame constriction at the patient's head and neck. Moreover, the frameless system allows imaging acquisition the day before the surgery and thus may reduce the time required for the procedure on the day of surgery (17).

The accuracy of frameless techniques compared with frame-based procedures has already been described elsewhere (18–20).

Previously, two clinical trials have evaluated the efficacy of frameless DBS surgery in Parkinson's disease patients showing similar outcomes when compared with frame-based stereotaxy (17, 21).

However, the effect of frameless surgery has not yet been assessed in dystonic patients, and clinical trials comparing the effects of frame-based DBS with those of a frameless approach are still lacking. This study aims to evaluate the clinical outcome of GPi-DBS in a homogeneous population of dystonic patients who underwent GPi-DBS surgery using a frameless technique or a frame-based stereotaxy.

## Materials and Methods

We performed a retrospective analysis of 20 patients (13 females and 7 males) affected by refractory primary generalized or multi-segmental dystonia who underwent bilateral GPi-DBS lead's implant between January 2008 and December 2012 in one single center (Udine's Hospital, Italy). Only two neurosurgeons were involved in OR, and each of them had a great experience in DBS frame-based stereotaxy since 2000 and, for frameless technique, more than 6 years at the time of collecting data.

According to our local regulations, this data collection (retrospective and on clinical charts) did not require any approval by the ethics committee. Ten consecutive patients underwent surgery with a frame-based approach (F group, where F stands for frame), whereas the other ten consecutive patients were implanted using a frameless technique (FL group, where FL stands for frameless).

The recruitment (randomization) was determined based on the availability of the stereotactic surgical technique: from 2008 to 2010, only a frame-based system was available in our center, whereas, since 2010, a frameless system was adopted.

All the patients included in this data collection were affected from severe dystonia, causing marked disability in their daily living activities. Patients were addressed to surgery if they met the following inclusion criteria: a primary generalized or multi-segmental dystonia, a normal psychiatric and cognitive profile, a normal brain magnetic resonance imaging (MRI) and lack of response to medical treatment including anticholinergics, benzodiazepines, neuroleptics, baclofen, and/or botulinum toxin injections.

After giving their written informed consent for surgery and data collections, the patients underwent bilateral implantation of a quadripolar electrode (Medtronic 3387-40®, Medtronic®, Minneapolis, MN) in the GPi and with an Activa-RC device in a single day.

### Frame-Based Sterotactic Surgery

The F group underwent stereotactic frame-based surgery (Leksell). Preoperative, non-stereotactic MRI scans (T1 with gadolinium and T2; slice thickness: 1.5 mm; without gap or overlap) were performed a day before the operation.

On the day of surgery, the Leksell frame was placed parallel to the intercommissural line. Immediately after, a stereotactic computed tomographic (CT) scan was obtained with 2-mm thick slices. The MRI datasets were then matched with the CT data (Medtronic Stealth Station Framelink® Medtronic, Minneapolis, MN). The target point (planning) was calculated indirectly by determining the midcommissural point on the T1-weighted MRI scan and was adjusted according to the T2-weighted MRI scan.

### Frameless Stereotactic Surgery

The FL group underwent frameless surgery by using the NexFrame system (Medtronic®, Minneapolis, MN). Preoperative, volumetric MRI brain scans (T1 with gadolinium and T2; slice thickness: 1.5 mm) were performed few days before the operation.

Then, the day before surgery, different metal fiducial markers were positioned on the skull bone, and a stereotactic CT cerebral scan was performed. Subsequently, the CT slices were matched with the MRI datasets (Medtronic Stealth Station Framelink® Medtronic, Minneapolis, MN). The target point (i.e., the ventroposterior lateral part of the GPi) was indirectly calculated by determining the midcommissural point on the T1-weighted MRI scan and was adjusted according to the T2-weighted MRI scan (direct visualization).

On surgery day, the patient was positioned on the operating table and the frameless system placed and fixed to the skull bone. The entry point on the skull and the trajectory planning to target have been based on the image's guidance (neuronavigation).

### Intraoperative Neurophysiologic Monitoring, Implantable Pulse Generator Implantation, and Post-operative Imaging

DBS surgery was done under local anesthesia for both groups. For all procedures, intraoperative neurophysiological monitoring with MER and macro-stimulation was performed.

MER was recorded by semi-microelectrodes (FC2002, Medtronic®, Minneapolis, MN) connected to the Leadpoint Workstation system (Medtronic®, Minneapolis, MN). MER recording started 10 mm above the calculated target and was performed on simultaneous five tracks on each side (anterior, central, posterior, medial, and lateral) in 1 mm step by using the *ben-gun Microdrive system*®. Semi-microelectrodes, with an impedance of at least 1 MΩ, were introduced by tubes placed in an array configuration for the F group and FL group.

An expert neurophysiologist performed qualitative intraoperative pattern discharge evaluation to identify “burster,” “pauser,” or high-frequency discharge neurons, as classically reported for globus pallidus neurophysiological patterns (22).

Intraoperative macro-stimulation test was performed at different depths in the track that displayed the richest cellularity during MER; standard stimulation parameters set-up where: length 100 μs, frequency 130 Hz, and intensity up to 3 mA. The definitive quadripolar lead (Medtronic mod. 3387-40®) was also implanted in this track unless the stimulation test reveals capsular or optic tract responses at lower current intensities.

To avoid displacement, all permanent leads were looked at by a burr-hole cap. On the same day, all the patients underwent a second-time surgery to place extension cables and insert implantable pulse generator (in the right subclavear region) under general anesthesia. To verify the final lead position within the GPi and exclude hemorrhagic events ongoing, we performed a postoperative cerebral CT scan on the same day or after 24 h from surgery. Postoperative CT data were matched with the preoperative MRI data by a Medtronic Stealth Station software (Framelink®, Medtronic, Minneapolis, MN); the lead position was detected in x, y, and z coordinates, and data were collected.

### Deep Brain Stimulation Programming and Clinical Evaluation

Stimulation was switched on 2 weeks after the lead implantation. At each follow-up visit, stimulation parameters were gradually modified and adapted according to the clinical benefit and/or the appearance of adverse effects. The pulse width and the voltage/current were gradually adapted according to the clinical evaluation (improvement of symptoms and/or appearance of collateral adverse effects). Monopolar cathodic stimulation was the final configuration in all the patients, and the frequency was not modified after 6 months. No modification of the pharmacological treatment was made after surgery.

A neurologist expert in movement disorders and in the DBS management scored the Burke–Fahn–Marsden Dystonia Rating Scale (BFMDRS) for each patient at baseline and 6 and 12 months after the lead implantation. Clinical assessments were always blinded and done by watching the video-recorded BFMDRS, according to a standardized protocol (23), without any knowledge of the stimulation setting adopted and after the acquisition of the informed consent of the patient to video recording.

The BFMDRS video recording was performed by a different neurologist, unblinded, who proceeded to the réglage of the stimulation parameters as usual in clinical practice. Follow-up assessment and setting of the stimulation parameters were always done by a neurologist expert in movement disorders at all times.

### Data Analysis

Clinical outcome (BFMDRS motor and disability scores variation), stimulation parameters, and demographic and clinical data were compared between the two groups of patients (F group *vs*. FL group) and within each group. *Post-hoc* comparisons were carried out when analysis of the variance was significant, to compare data two by two, applying the Bonferroni correction, with significance at p < 0.05 as an adjustment for multiple comparisons.

Non-parametric tests were used to compare clinical data. The variation over repeated measurements was assessed by means of the Friedman test. The comparison between the two groups was performed by means of the Wilcoxon–Mann–Whitney test (*p* < 0.05 was considered statistically significant). Statistical analyses were performed using SPSS 12.0 (SPSS, Inc., Chicago, Ill., USA).

Each patient provided informed consent to surgery and to use their clinical data. Our local ethics committee approved this observational, retrospective, non-profit study (80/2013/Sper/CERU).

## Results

Twenty patients with early or late-onset refractory primary dystonia were included in the study.

The ages at surgery were as follows: 46 ± 11.17 years (mean ± SD)—range 21–63 years—for the F group and 41.3 ± 12.71 years (mean ± SD)—range 23-66 years—for the FL group.

The disease durations were as follows: 22.2 ± 12.71 years (mean ± SD)—range 5–39 years—for the F group and 23.3 ± 11.83 years (mean ± SD)—range 2–40 years—for the FL group. No statistical difference was found in the demographical data between the two groups (*p* = 0.3060 for age at surgery, *p* = 0.8797 for disease duration).

Among the two groups, a postoperative evaluation of the lead's position did not evidence any statistical differences in x, y, or z coordinates (mean values and SD were as follows: X = 20.20 ± 1.64, Y = 3.09 ± 1.29, Z = 3.26 ± 1.06 for the F group and X = 20.40 ± 1.77, Y = 3.15 ± 1.57, Z = 3.60 ± 1.78 for FL group).

**Figure 1:**
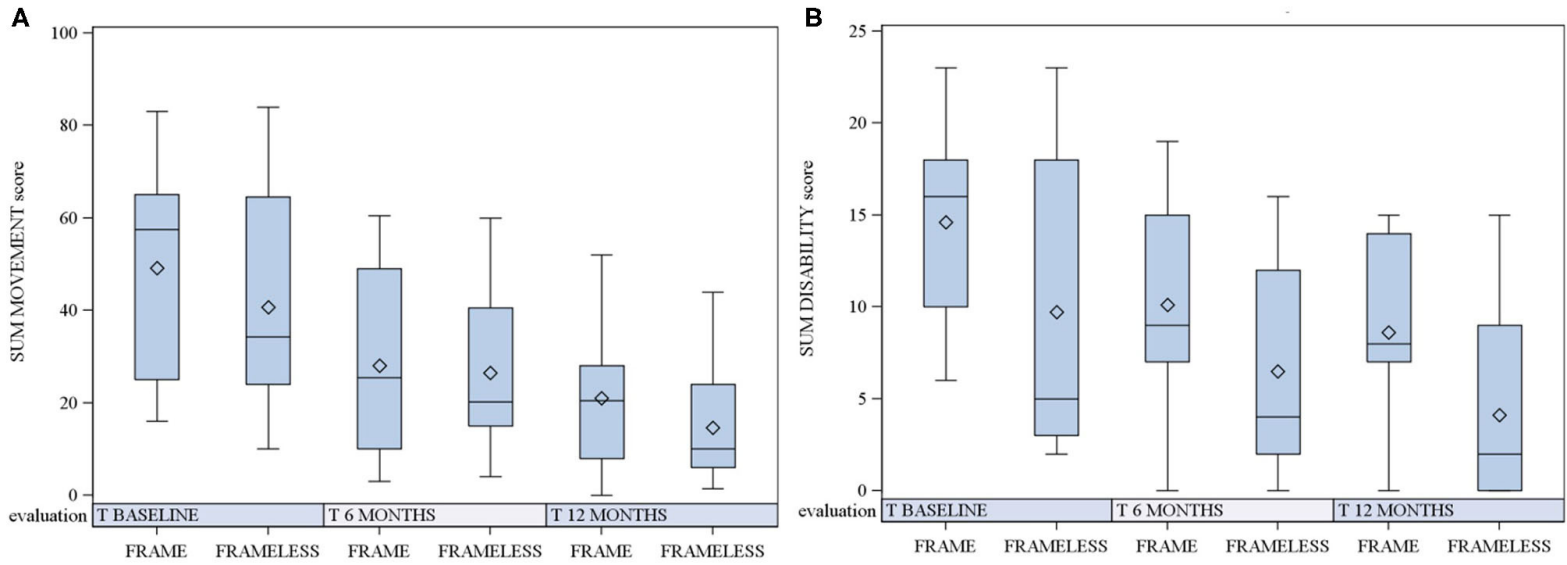
BFMDRS scores in the frame (F) and frameless (FL) groups at baseline and 6 and 12 months after surgery. Scores in boxplots are described as mean, median and 25 and 75th percentile. Comparison between the two groups is reported. **(A)** BFMDRS-M (motor scores); **(B)** BFMDRS-D (disability scores).

Detailed results of the BFMDRS scores are summarized in Table 1 and Figure 1.

**Table 1:** Demographic data and Burke–Fahn–Marsden Dystonia Rating Scale (BFMDRS), Movement, and Disability scores in the frame and frameless group at baseline and 6 and 12 months after surgery.

		**Age at** **surgery**	**Disease duration**	**BFMDRS** **baseline**		**BFMDRS** **6 months**		**BFMDRS** **1 year**		***P-value*** **BFMDRS-M baseline/6 months/1 year**	***P-value*** **BFMDRS-D baseline/6 months/1 year**
				**M**	**D**	**M**	**D**	**M**	**D**		
FRAMELESS (FL) 10 patients	Mean	41	23.3	40.70	9.7	26.5	6.5	14.65	4.10	<0.0001	<0.0001
	± SD	12.71	11.83	25.53	8.21	18.11	5.70	13.62	5.34		
	Median	-	-	34.25	5	20.25	4	10	2		
	25th	-	-	24	3	15	2	6	0		
	75th	-	-	64.5	18	40.50	12	24	9		
FRAME (F) 10 patients	Mean	46	22.2	49.20	14.60	28.10	10.10	21	8.6	<0.0001	<0.0001
	± SD	11.1	12.71	23.33	5.39	20.02	5.93	16.03	5.12		
	Median	-	-	57.5	16	25.50	9	20.50	8		
	25th	-	-	25.75	10	10	7	8	7		
	75th	-	-	65	18	49	15	28	14		
*P-value*	FL vs. F	0.3060	0.8797	0.5450	0.1379	0.7912	0.1974	0.4053	0.1086	-	-

*Statistical comparison between the two groups of patients (below the table) and inside each group (last columns) are reported*.

In the F group, the BFMDRS-M score at baseline was 49.20 ± 23.33 (mean ± SD; median 57.5, 25th percentile 25, 75th percentile 65); the BFMDRS-D score at baseline was 14.60 ± 5.39 (mean ± SD; median 16, 25th percentile 10, 75th percentile 18). After 6 months, the BFMDRS-M score was 28.10 ± 20.02 (mean ± SD; median 25.50, 25th percentile 10, 75th percentile 49) with a 43% of mean improvement in comparison with baseline; the BFMDRS-D score was 10.10 ± 5.93 (mean ± SD; median 9, 25th percentile 7, 75th percentile 15) with a 30.82% of mean improvement. After 12 months, a further improvement occurred for both the movement and disability scale, respectively: 21 ± 16.03 (mean ± SD; median 20.50, 25th percentile 8, 75th percentile 28) for BFMDRS-M (mean improvement of 57.31% in comparison with baseline) and 8.6 ± 5.12 (mean ± SD; median 8, 25th percentile 7, 75th percentile 14) for BFMDRS-D (mean improvement of 41.10% in comparison to baseline).

In the FL group, the BFMDRS-M score at baseline was 40.70 ± 25.53 (mean ± SD; median 34.25, 25th percentile 24, 75th percentile 64.5); the BFMDRS-D score at baseline was 9.7 ± 8.21 (mean ± SD; median 5, 25th percentile 3, 75th percentile 18).

After 6 months, the BFMDRS-M score was 26.5 ± 18.11 (mean ± SD; median 20.25, 25th percentile 15, 75th percentile 40.50) with a 34.88% of mean improvement in comparison with baseline; the BFMDRS-D score was 6.5 ± 5.70 (mean ± SD; median 4, 25th percentile 2, 75th percentile 12) with a 33% of mean improvement. After 12 months, patients improved further as follows: 14.65 ± 13.62 (mean ± SD; median 10, 25th percentile 6, 75th percentile 24) for BFMDRS-M with a mean improvement of 64% and 4.10 ± 5.34 (mean ± SD; median 2, 25th percentile 0, 75th percentile 9) for BFMDRS-D with a mean improvement of 57.73% in comparison with baseline.

The F group and FL group did not show any significant difference in the severity of BFMDRS scores at baseline (*p* = 0.5450 for motor score and *p* = 0.1379 for disability score).

Similarly, as shown in Figure 1, no statistically significant difference was found both at 6 months (*p* = 0.7912 for motor score and *p* = 0.1974 for disability score) and at 12 months after surgery (*p* = 0.4053 for motor score and *p* = 0.1086 for disability score).

No surgical complication occurred in both groups, and even if the GPi target is more lateral than in the case of subthalamic nucleus or ventral intermediate nucleus targeting, we did not have any limitations in the use of the NexFrame system®.

The stimulation parameters activated in the two groups at 6 and 12 months are reported in Table 2. As shown, there was not any statistically significant difference between the two groups.

**Table 2:** Stimulation parameters adopted in our dystonic patients at 6 and 12 months after surgery.

	**6 months after DBS surgery**		**1 year after DBS surgery**		***P-value 6 months vs. 1 year stimulation parameters***	
	**Voltage (V)**	**PW (μs)**	**Voltage (V)**	**PW (μs)**	**Voltage**	**PW**
	**Mean ± SD**	**Mean ± SD**	**Mean ± SD**	**Mean ± SD**		
Frame	2.19 ± 0.46	132.5 ± 20.72	2.93 ± 0.85	157.5 ± 45.41	0.0195^*^	0.0625
Frameless	2.31 ± 0.53	138.4 ± 38.43	2.51 ± 0.47	159.7 ± 36.86	0.0156^*^	0.0313^*^
*P-value*	0.8498	0.9034	0.1984	0.6952	-	-
Frame vs. Frameless						

*Statistical comparison between the two groups of patients (below the table) and inside each group (on the right) are reported*.

*PW, pulse width*.

* *p = 0.05*.

## Discussion

Frame-based stereotactic procedures have historically been the gold standard for precise and accurate targeting of deep brain structures. With the target-centered arc of the Leksell frames, the axis of movement along the arc always has a trajectory to the target point (19). This allows for minor modifications of the trajectory intraoperatively without requiring recalibration of the entire frame system. Frame systems for stereotactic implantation of DBS electrodes can raise certain adversities to patients, surgeons, and anesthesiologists. Patients may have difficulty tolerating the frame during a procedure that severely limits the range of motion, lasts several hours, and requires active participation. These systems require that a CT or MRI scan should be taken immediately after placement of the frame. Frame placement, image acquisition and registration, target planning, and then translation of coordinates for the frame significantly increase the surgical time and patient discomfort but also pledges surgery resources and time (24).

In recent years, frameless stereotaxy has been developed to reduce patient discomfort and to improve the ability to perform a neurological evaluation during DBS surgery while keeping the same high accuracy in DBS lead placement (18, 19).

The overall surgical procedure for implantation of DBS with frameless systems is similar to those of the frame-based procedure. However, instead of fixing the head to a rigid frame that prevents head motion, a lighter-weight, frameless system is fixed to the head and moves with it. The patient's head is usually held rigidly in place with a Mayfield head-holder, and the fiducials reference points are registered to the preoperative imaging with a hand-held probe and infrared camera (24, 25).

Although frameless image guidance has become a standard technique for open craniotomies such as tumor resection, the accuracy of such systems was not considered sufficient for trajectory-based procedures such as lesioning or stimulation of deep nuclei (24).

A study published by Henderson et al. using bone fiducials on a phantom skull, demonstrated a mean localization error of 1.25 mm, which compares favorably with localization errors published for the CRW (1.8 mm) and Leksell (1.7 mm) frames (26).

The accuracy of frameless systems in a clinical picture has been described in several studies: Holloway et al. (18) evaluated final DBS lead positions using postoperative CT scan in 38 patients who were implanted using a skull-mounted trajectory guide and an image-guided workstation. Multivariate analysis of variance demonstrated no statistically significant difference in the accuracy of the two methods. Bjartmarz and Rehncrona (19) compared the accuracy and precision of frameless neuronavigation with conventional frame-based stereotaxy during bilateral ventral intermediate nucleus DBS in 14 patients with essential tremors. The comparison reveals better accuracy of the frame-based technique; with proper attention to factors such as fiducial identification, registration, system accuracy, and platform stability, frameless techniques can equal or exceed the accuracy of stereotactic frames (26, 27).

Recently, the clinical outcome of frame-based surgery in parkinsonian patients has been compared with that of frameless techniques in two studies: Bronte-Stewart et al. (21) evaluated 31 subjects affected by Parkinson's disease who underwent subthalamic nucleus DBS with a frameless approach. By means of the Unified Parkinson's Disease Rating Scale III, a 58% improvement with a mean reduction in medication of 50% was found. This result is consistent with the published outcomes using the frame-based technique.

Tai et al. (17) described 24 patients with advanced Parkinson's disease operated on with subthalamic nucleus stimulation, 12 with frameless and 12 with frame-based stereotaxy. After 1 year of follow-up, the patients who received frameless surgery showed no difference in the degree of improvement in clinical motor function compared with the patients who received frame-based surgery (*P* = 0.819); the average improvement rates were 60.9 and 56.9%, respectively, in the stimulation alone/medication-off state, as evaluated by the Unified Parkinson's Disease Rating Scale III motor subscore. However, the FL group had a significantly shorter total MER time (*P* = 0.0127) and a smaller number of trajectories (*P* = 0.0096) than the F group.

Recently, we confirmed (20) the accuracy and precision of the frameless system in different DBS surgeries by studying 110 subjects (220 targets) and demonstrating that the frameless system is reliable. In that study, the mean error in lead's position in coordinates was calculated in 60 GPi nuclei by considering the differences between the intraoperative lead location targeted by electrophysiology and the final lead location. The differences detected were of 0.75 mm (SD 0.44) for x-axis, 0.73 mm (SD 0.42) for y-axis, and 0.77 mm (SD 0.41) for z-axis.

Our study proved the efficacy of pallidal stimulation in the treatment of primary dystonia, as previously described by other authors (1, 3), and we did not detect any statistical difference of clinical outcomes between the FL and F surgery groups after 6 and 12 months of follow-up. To our knowledge, this is the first report describing the clinical outcome of GPi-DBS in dystonia using a frameless surgical approach.

Even if the main goal of this study is not to compare two different surgical techniques, which are also related to the experience and preferences of each neurosurgeon, the frameless technique offers some advantages when compared with frame-based surgery in dystonia: imaging and trajectory planning can be performed before the day of surgery, reducing operating room time. Without a frame, patients are able to move their heads and reposition themselves during surgery, which provides increased comfort and compliance; the lack of the frame enables the examiner to observe facial features during test stimulation, to communicate easier to the patient, and in case of general anesthesia, the assistant is easier.

Nevertheless, some critical points during frameless surgery must also be considered: the entry point detection, that it could be influenced by the shape of the skull (28), the use of a neuroimaging navigation system, and, finally, higher costs for the system (17, 24).

Notably, in our experience regarding GPi-DBS, the limited range of angulation using Nexframe frameless system should be taken into account, but it does not preclude any limitation in the parasagittal approach to the target also subjects with skull curvature, although the use of a neuroimaging navigator system is now largely diffuse in neurosurgery. Finally, the higher cost for the frameless system is decreasing over time, and the surgeon's team became familiar with the application of this technology, improving the surgery time of DBS surgery in comparison with the frame-based GPi-DBS.

This study confirms the efficacy and safety of frameless stereotaxy in dystonic patients during GPi-DBS surgery as well. Nevertheless, it has some limitations, that is, the relatively small number of patients involved, the retrospective design, and the short follow-up period. Considering the lack of randomization, one possible criticism of our study could be the dishomogeneity of the population involved: however, the lack of statistically significant difference between the demographic data and BFMDRS scores at baseline does reveal the homogeneity of statistic samples. Therefore, clinical outcome was not influenced by possible different stimulation settings, as we used comparable parameters in both groups. These features make our study more reliable and significant.

## Conclusions

GPi-DBS is an effective and well-established treatment for dystonia, both with a frame-based and a frameless approach with comparable clinical outcomes and surgical complications. However, frameless techniques could be a better choice considering the minimal discomfort to the patients and the shorter operating room time needed as compared with frame-based approaches, even if larger randomized controlled trials are needed to compare these different surgical procedures to conclude which one may or may not be better.

## Data Availability Statement

The raw data supporting the conclusions of this article will be made available by the authors, without undue reservation.

## Ethics Statement

The studies involving human participants were reviewed and approved by Comitato Etico Udine (CERU). Code # 80/2013/Sper/CERU. The patients/participants provided their written informed consent to participate in this study.

## Author Contributions

RE, SR, GD, and CL designed and projected the full study. All the authors collected information and providing data about the clinical follow-up of the subjects. SR, CL, and NG reviewed the clinical scales. RE, SR, and GD wrote the paper while CL, MM, SD'A, NG, and MS reviewed and suggested integrations on manuscript.

## Conflict of Interest

The authors declare that the research was conducted in the absence of any commercial or financial relationships that could be construed as a potential conflict of interest.
